# MiR-99a may serve as a potential oncogene in pediatric myeloid leukemia

**DOI:** 10.1186/1475-2867-13-110

**Published:** 2013-11-05

**Authors:** Lidan Zhang, Xiaojuan Li, Zhiyong Ke, Libin Huang, Yanni Liang, Jun Wu, Xiaoli Zhang, Yueqin Chen, Hua Zhang, Xuequn Luo

**Affiliations:** 1 Department of Pediatric, The First Affiliated Hospital of Sun Yat-Sen University, Zhongshan Er Lu, Guangzhou 510080, China; 2 Key Laboratory of Gene Engineering of the Ministry of Education, State Key Laboratory for Biocontrol, Sun Yat-Sen University, Guangzhou 510275, China; 3 China-America Cancer Research Institute, Key Laboratory for Medical Molecular Diagnostics of Guangdong Province, Guangdong Medical College, 1 Xincheng Road, Song-Shan Lake (SSL) Science, Technology and Industrial Park, Dongguan, Guangdong 523808, China

**Keywords:** miR-99a, CTDSPL, TRIB2, Myeloid leukemia, Childhood

## Abstract

**Background:**

Leukemia is the most common malignant proliferative disease in children. Our previous study found that miR-99a was up-regulated in pediatric primary AML using microRNA expression profiles. Up to date, although there is a certain number of reports on microRNA expression features and functions in pediatric acute myeloid leukemia (AML) and chronic myeloid leukemia (CML), the expression and function of miR-99a in these diseases remain to be investigated.

**Methods:**

qRT-PCR was performed to measure the expression level of miR-99a in 88 samples including 68 pediatric acute myeloid leukemia patients, 8 chronic myeloid leukemia patients and 12 pediatric controls. MTT assay, apoptosis assay, dual-luciferase reporter transfection assay and western blot analysis were used to investigate the function of miR-99a.

**Results:**

MiR-99a was highly expressed in pediatric-onset AML (M1-M5) and CML, while significantly lowly expressed during complete remission of these diseases. MTT assay indicated that the proliferations of K562 and HL60 cells were significantly promoted by miR-99a, and apoptosis assessment by Annexin V/propidium iodide staining demonstrated that the apoptosis of these cells was inhibited by miR-99a. Additionally, dual-luciferase reporter transfection assay and western blot analysis indicated that miR-99a may target CTDSPL and TRIB2, which are two tumor suppressor genes.

**Conclusions:**

This study revealed that miR-99a may play a potential oncogenic role in pediatric myeloid leukemia including AML and CML via regulating tumor suppressors CTDSPL and TRIB2, suggesting that these two leukemias might share some common biological pathways involved in the generation and development of disease and miR-99a could be a common therapeutic target for myeloid leukemias treatment.

## Background

Leukemia is one of the leading causes of cancer death worldwide. Acute myeloid leukemia (AML), one type of malignant diseases, arises from myeloid progenitor cells that are arrested at early stages of differentiation. Chronic myeloid leukemia (CML) is a clonal disorder in which cells of the myeloid lineage undergo massive clonal expansion. Although the recent advancement in understanding and treatment of AML and CML has remarkably improved the cure rate over the past decade, a number of patients still die of these diseases. This highlights the need for more thorough knowledge of these two leukemias.

Recently, microRNAs (miRNAs), a class of non-coding RNA, were found to play important roles in various fundamental biological processes, such as cell proliferation, apoptosis, differentiation and signaling pathway, which are accomplished by silencing specific target genes through translational repression or direct mRNA degradation [1-3]. Studies demonstrated that about 50% of annotated human miRNAs are located at fragile sites and genomic regions involved in cancers [4]. Some miRNAs are involved in cancer regulation and are considered as oncogenes or tumor suppressors [5]. The expression profiles of miRNAs could be linked to disease diagnosis, therapeutic response and prognosis [6-10]. The first finding linking miRNAs and leukemia was that adult patients with chronic lymphocytic leukemia often have deletions or downregulation of miR-15 and miR-16 at 13q14 [11]. Up to date, an increasing number of studies have revealed that the differentiation of AML lineages is regulated by miRNAs, which have key roles in hematopoiesis [12,13].

Our previous miRNA profiling analysis showed the expression of miR-99a in pediatric-onset AML (FAB M1-M3) was 3.81 times higher at average than that in normal donors [14], suggesting miR-99a may be involved in the progression of pediatric myeloid leukemia. To confirm this, we performed further investigation to assess the expression of miR-99a in childhood AML and CML, and the function of miR-99a in these diseases.

## Methods

### Clinical samples

A total of 88 bone marrow samples were enrolled in this study. The samples taken by bone marrow puncture were from 68 pediatric acute myeloid leukemia patients (including 41 samples before therapy, 23 samples with complete remission and 4 samples with relapse), 8 chronic myeloid leukemia patients (including 4 samples before therapy and 4 samples with complete remission) and 12 pediatric controls from the First and Second Affiliated Hospital of Sun Yat-Sen University. The newly diagnosed AML patients included 6 with M1, 17 with M2, 10 with M3, 4 with M4 and 4 with M5. The AML patients with complete remission (CR) included 1 with M1, 6 with M2, 6 with M3, 6 with M4 and 4 with M5. The relapsed AML patients were 4 with M2. Patients^’^ characteristics are shown in Additional file 1: Tables S1 and S2. Written informed consent for biological studies was obtained and the study was approved by the Ethics Committee of the affiliated hospitals of Sun Yat-Sen University.

### Cell culture and RNA/protein isolation

Human HL60 (acute myeloid leukemia cell line) and K562 cells (chronic myeloid leukemia cell line) were cultured in RPMI 1640 medium (Invitrogen). HEK-293 T, the human embryonic kidney cell line, was grown in Dulbec-co’s modified Eagle’s medium (Invitrogen). Both cultures were supplemented with 10% fetal bovine serum (fetal bovine serum, Australia) and sodium pyruvate, and cultured at 37°C in a humidified atmosphere consisting of 5% CO_2_. Total RNA and protein were isolated from clinic samples with Trizol (Invitrogen, Carlsbad, CA) according to the instructions of the manufacturer.

### Quantitative real-time PCR analysis for miR-99a expression

Quantitative real-time reverse transcriptase PCR (qRT-PCR) was performed to detect miR-99a expression. Briefly, 0.2 μg of small RNA extracted from cell samples was reverse-transcribed to cDNA using M-MLV reverse transcriptase (Promega) and amplified with specific designed miRNA RT-primers and PCR amplification primers (Sangon, Shanghai, China). Sequences of all the primers are shown in Additional file 1: Table S3. The expression level of each miRNA was measured using the 2-DeltaDeltaCt method.

### MTT assay

K562 and HL60 cells were respectively plated at 1 × 10^4^ per well. The cells were transfected with 100 nM miR-99a mimics/NC (miR-99a/scrambled oligonucleotides) or inhibitor-miR-99a/NC using Lipofectamine 2000 (Invitrogen) following manufacturer’s recommendation and were then incubated for 24 h, 48 h and 72 h, respectively. Next, the cells were incubated with Dye Solution (15 μL) for another 4 h until purple precipitate was visible. Lastly, after 100 μL Stop Mix was added, the cells were left at room temperature in the dark for 2 h and the absorbance was recorded.

### Apoptosis assay

K562 and HL60 cells were transfected with miR-99a mimics/NC or inhibitor-miR-99a/NC (100 nM) using Lipofectamine 2000 as mentioned above. The cells were collected at 48 h, 72 h and 96 h post transfection respectively. The cells were centrifuged and resuspended in 500 μl of staining solution (containing annexin V fluorescein and propidium iodide in HEPES buffer) (annexin V: FITC apoptosis detection kit; BD PharMingen, San Diego, CA). After incubation at room temperature for 15 min, cells were analyzed by flow cytometry.

### Target genes prediction and vector constructs

The potential targets of miR-99a were predicted by means of TargetScan ( http://www.targetscan.org/) and PICTAR ( http://pictar.mdc-berlin.de/) software. In order to reduce the number of false positives, only putative target genes predicted by both programs were accepted. The vectors of pre-miR-99a and the potential targets predicted (TRIB2 and CTDSPL) were constructed. Briefly, the primers of miR-99a were designed to amplify pre-miR-99a by PCR from genomic DNA. The amplified products were ligated into the PCD6.2 vector (Promega, Madison, WI). Ecological forms and mutants of the potential targets of miR-99a, designed by TargetScan and generated by annealing, were ligated into the pGL3 vector or the psi-CHECK-2 vector (Promega, Madison, WI). Proper insertions were all confirmed by DNA sequencing. All the primers were synthesized (Sangon, Shanghai, China) and the information is available in Additional file 1: Table S4.

### Cell transfections and Luciferase assays

HEK-293 T cells were grown in 24-well plates at a density of 1 × 10^5^ cells per well in 0.5 ml of complete growth medium and allowed HEK-293 T cells to adhere overnight. K562 cells were grown in 24-well plates at a density of 1 × 10^6^ cells per well. 0.1 μg of pre-miR-99a and the potential targets vectors were transfected into HEK-293 T cells using Lipofectamine 2000 (Invitrogen) and were transfected into K562 cells by electroblotting (Ambion, Austin, Texas) respectively in growth medium according to manufacturer’s recommendation. After 24-48 h, the transfected cells were harvested for Dual-luciferase reporter transfection assay. Similarly, 100 nM miR-99a mimics/NC duplex or inhibitor/inhibitor-NC were used for transfection.

### Western immunoblotting

K562 and HL60 cells were treated as indicated in the figures and lysed in RIPA buffer (Pierce, Rockford, IL, USA) with protease and phosphatase inhibitors (Roche, Beijing, China). The protein of bone marrow, K562 and HL60 cells was quantified using the BCA protein assay (Pierce, Rockford, Illinois). Protein (30 μg) was loaded onto a 12% SDS–PAGE gel then transferred onto nitrocellulose. The membrane was blocked for 2 h in Tris-buffered saline Tween-20 (TBST) containing 2% bovine serum albumin, and cleaved parp was incubated with rabbit anti-CTDSPL (1:10000, Cell Signaling, Danvers, MA) and mouse anti-TRIB2 (1:5000, Abcam, Invitrogen, USA) overnight at 4°C. After incubation with HRP-conjugated secondary anti-mouse or anti-rabbit (ABR, Golden, Colorado) at room temperature for 1 h, blots were then developed according to ECL Substrate (Pierce) following manufacturer’s instructions. Protein was normalized with β-actin (Sigma St. Louis, Missouri), Tublin (Cell Signaling Technology, USA) and GAPDH (Cell Signaling Technology, USA), and measured by densitometry by two independent researchers.

### Statistical analysis

T test, ANOVA and non-parametric rank sum test were performed using SPSS16.0 statistical software. A Fisher r-to-z transformation was carried out to calculate a probability level (*P* value). Student’s t test was performed to assay the statistical significance.

## Results

### MiR-99a is highly expressed in pediatric AML and CML at diagnosis, while significantly lower expressed during complete remission of the diseases

**Figure 1:**
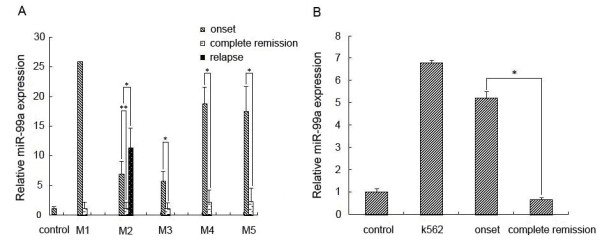
**The expression of miR-99a assessed by real-time qRT-PCR in pediatric AML (M1-M5) (A) and CML (B) patients before and after therapy.** The data were normalized against U6 expression in the same patient. *P < 0.05, **P < 0.01. There was only one patient with AML-M1 available for evaluation and thus no statistical analysis was performed.

To investigate miR-99a expression in different subtypes and disease stages of pediatric AML, qRT-PCR was performed on 62 bone marrow samples including 12 pediatric controls, 23 newly diagnosed (only 23 samples of 41 newly diagnosed AML patients available due to poor quality or limited amouts of RNA), 4 relapse and 23 CR patients, all of which were not in pairs. The 23 newly diagnosed patients included 2 with M1, 7 with M2, 6 with M3, 4 with M4 and 4 with M5. Results showed that miR-99a was highly expressed in all of the onset patients with M1 to M5, however, in 91% of the CR patients, miR*-*99a expression decreased sharply to a level similar to that in normal controls (Figure 1A). In addition, the expression of miR-99a in the relapsed patients with M2 increased obviously, and was even higher than that of their onset counterpart (Figure 1A).

For CML, a total of 20 bone marrow samples including 12 pediatric controls and 8 CML children (4 at newly diagnosis and 4 in CR), were also used for qRT-PCR. The results displayed that miR-99a expression significantly increased in all cases at diagnosis (median 5.21), while distinguishedly decreased in those during CR (median 0.71). The former was 7.34 times of the latter (Figure 1B). Moreover, qRT-PCR revealed the expression of miR-99a in K562 cells, a CML cell line, was approximately 9.54 times of that in CR patients. Therefore, it was inferred that miR-99a might be involved in proliferation and apoptosis of myeloid leukemia.

### MiR-99a promotes the proliferation and inhibits the apoptosis of HL60 and K562 cells

In order to further elucidate and prove whether miR-99a may function as an oncogene in pediatric AML and CML, the proliferation of HL60 and K562 cells was measured by MTT assay when miR-99a was overexpressed or downexpressed respectively.

**Figure 2:**
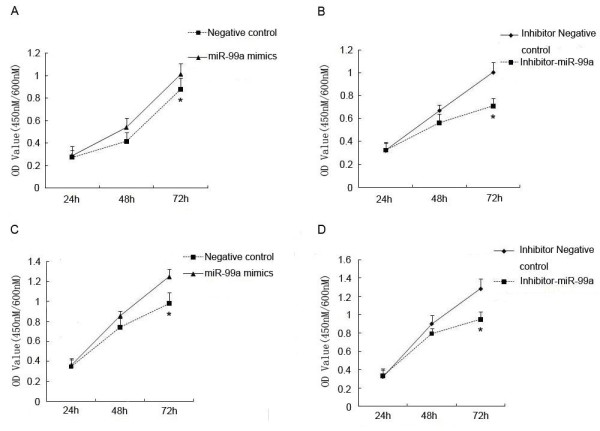
**MiR-99a promotes the proliferation of HL60 and K562 cells.** HL60 and K562 cells were transfected with miR-99a mimics, inhibitor-miR-99a or corresponding negative control respectively. MTT assay shows miR-99a significantly promotes the proliferation of the HL60 **(A)** and K562 **(C)** cells, and inhibitor-miR-99a reduces the proliferation of the HL60 **(B)** and K562 **(D)** cells. *P < 0.05.

HL60 and K562 cells were transfected with miR-99a or a random sequence of non-mammalian miRNA chain (negative control, NC), and were cultured for 24 h, 48 h, 72 h and 96 h, respectively. The survival rates of HL60 and K562 cells were then measured at 570 nm. Each experiment was repeated for four times. Figure 2A and 2C showed that cell survival rates in miR-99a groups are significantly higher than those in NCs and reach a peak after culture for 72 h. The proliferation of HL60 and K562 cells was stimulated by the overexpression of miR-99a, and this effect was time-dependent. Meanwhile, the survival rates of HL60 and K562 cells transfected with inhibitor-miR-99a were significantly decreased (Figure 2B and 2D). These suggest that miR-99a can promote the proliferation of HL60 and K562 cells. In addition, to check the transfection efficiency, labeled negative control was transfected into HL60 and K562 cells respectively under the same conditions. Additional file 2: Figure S1 demonstrates high transfection efficiency at 36 h after transfection.

**Figure 3:**
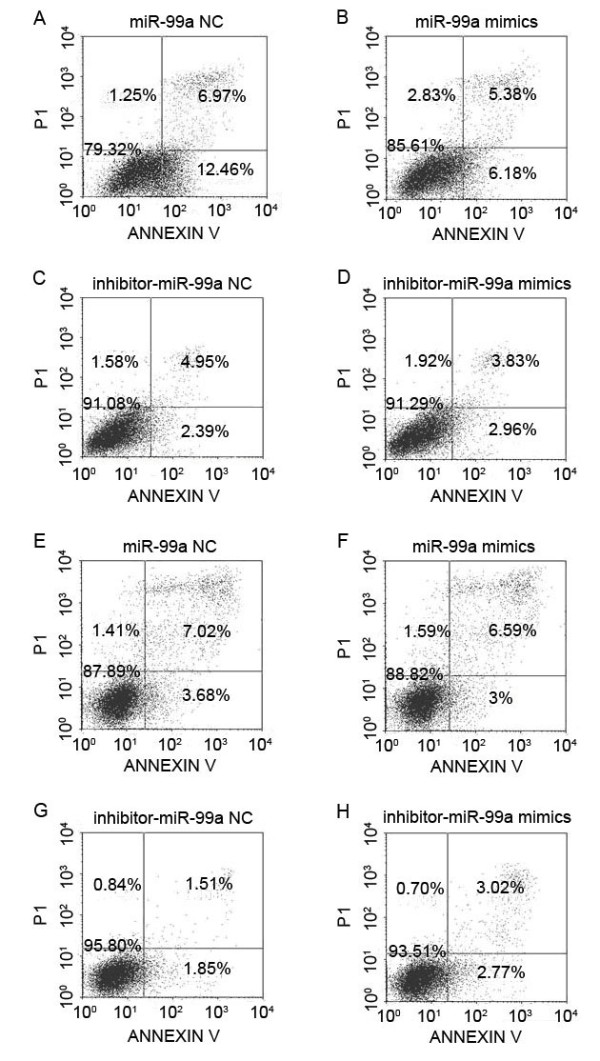
**MiR-99a inhibits the apoptosise of HL60 and K562 cells.** Apoptosis assessment by Annexin V/propidium iodide staining shows the apoptotic rates of K562 and HL60 cells transfected with miR-99a mimics, inhibitor-miR-99a or corresponding NC respectively. The fourth quadrant represents apoptotic cells detected by flow cytometry. **(A, B)** Of the K562 cells measured at 72 h after transfection, miR-99a inhibits apoptosis and the inhibition rate is 50.3%. **(A)** miR-99a NC, **(B)** miR-99a mimics. P < 0.05. **(C, D)** Of the K562 cells at 48 h, inhibitor-miR-99a prevents the inhibition of apoptosis and the prevention rate is 24%. **(C)** inhibitor-miR-99a NC, **(D)** inhibitor-miR-99a mimics. P > 0.05. **(E, F)** Of the HL60 cells at 96 h, miR-99a inhibits apoptosis and the inhibition rate is 18.5%. **(E)** miR-99a NC, **(F)** miR-99a mimics. P > 0.05. **(G, H)** Of the HL60 cells at 48 h, inhibitor-miR-99a prevents the inhibition of apoptosis and the prevention rate is 50%.** (G)** inhibitor-miR-99a NC, **(H)** inhibitor-miR-99a mimics. P < 0.05.

Since the study above suggested that miR-99a could promote the proliferations of HL60 and K562 cells, we then further assessed whether miR-99a could suppress the apoptosis of HL60 and K562 cells. Apoptosis assessment by Annexin V/propidium iodide staining was performed. HL60 and K562 cells were collected at 48 h, 72 h and 96 h were analyzed by flow cytometry, respectively. As expected, Figure 3 showed that the apoptotic rates of both HL60 and K562 cells are lower in miR-99a groups and higher in inhibitor-miR-99a groups when compared with their corresponding NCs, although significant difference of the apoptotic rates were observed only in K562 cells between miR-99a and NC groups, and in HL60 cells between inhibitor-miR-99a and NC groups, respectively.

Taken together, these data indicated that miR-99a might function as a potential oncogene and contribute to pediatric AML and CML progression by promoting proliferation and inhibiting apoptosis of myeloid leukemic cells.

### MiR-99a targets CTDSPL and TRIB2

**Figure 4:**
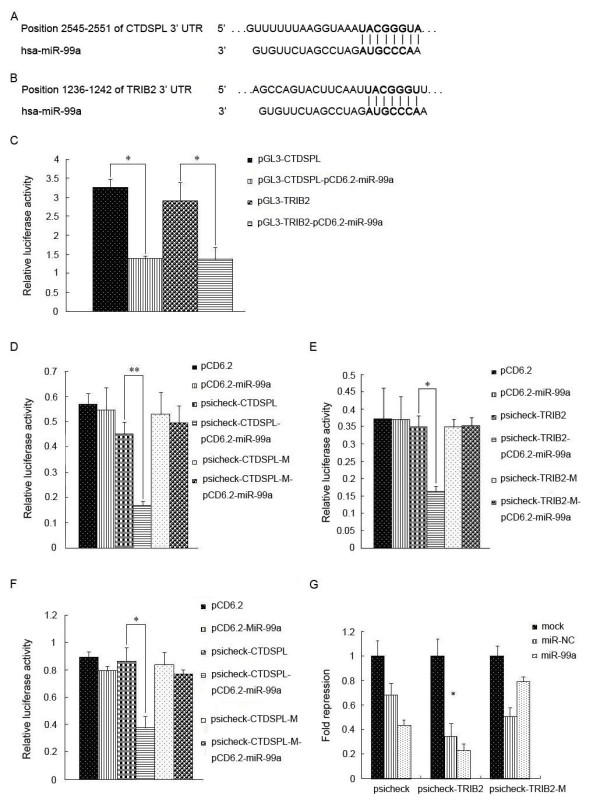
**MiR-99a Target genes prediction, selection and verification. (A, B)** The putative miR-99a target sequences in CTDSPL and TRIB2 genes respectively. **(C)** When CTDSPL or TRIB2 cloned into pGL3 vector was co-transfected into HEK-293 T cells with miR-99a precursor which had been cloned into pCD6.2 vector, luciferase activity was suppressed by 51-53% or 45-49% respectively. **(D, E)** MiR-99a target genes verification. Both the wild type and the full mutation of CTDSPL and TRIB2 were synthesized. When the wild-type of CTDSPL or TRIB2 was co-transfected with miR-99a precursor into HEK-293 T cells, luciferase activity was suppressed by 58-61% or 53-54% respectively. **(F, G)** Further verification of miR-99a Target genes (CTDSPL and TRIB2) through luciferase reporter assay in K562 cells by electroporation. Error bars represent standard deviation and each data was obtained from three independent experiments. *P < 0.05. **P < 0.01.

As we know, a large number of studies have shown that miRNAs play roles through their downstream target genes. In order to reduce the number of false positives, only putative target genes predicted by both programs were accepted. Among them, CTDSPL and TRIB2 are closely associated with leukemia cell apoptosis and were chosen to be further validated in HEK-293 T cells using luciferase reporter assays. Figure 4A and 4B show that the predicted miR-99a-binding sites in 3’UTR of CTDSPL and TRIB2 .

CTDSPL, the protein of carboxy-terminal domain RNA polymerase II polypeptide A small phosphatase (CTDSP) family, is a recently identified phosphatase-like tumor suppressor gene [15]. A current research showed that CTDSPL relates to the regulation of cell growth and differentiation, and frequent mutations or deregulation of this gene are disclosed in human hematopoietic cell and myeloid leukemia cell lines [16,17]. TRIB2 has 3 distinct regions, proline-rich N-terminus, serine/threonine kinase homology domain and C-terminal constitutive photomorphogenesis 1 (COP1)-binding domain. It acts as either a tumor suppressor or a cancer promoter in different biological conditions. We observed that when the wild-types of CTDSPL and TRIB2 were respectively co-transfected with miR-99a precursor into HEK-293 T cells, their luciferase activities were obviously suppressed by 51-53% and 45-49%, respectively (Figure 4C). Therefore, CTDSPL and TRIB2 may be the target genes of miR-99a.

Furthermore, the wild-type of CTDSPL or TRIB2, or the mutations of CTDSPL or TRIB2, deleting the seed sequence, was co-transfected with miR-99a precursor into HEK-293 T cells respectively. Results showed that the miR-99a precursor suppressed the luciferase activity of the wide type CTDSPL by 58-61% and the luciferase activity of the wide type TRIB2 by 53-54%. However, the full mutations of CTDSPL and TRIB2 abrogated the repressive ability of miR-99a, demonstrating the specificity of miR-99a target sequence in CTDSPL and TRIB2 (Figure 4D and 4E). As shown in Additional file 2: Figure S2, we can observe that HEK-293 T cells maintained in good condition and displayed high transfection rate, indicating the high accuracy and reliability of the experimental results.

CTDSPL and TRIB2 were preliminarily confirmed as two target genes of miR-99a in HEK-293 T cells. In K562 cells, a leukemic line, further verification experiments were carried out. The results were consistent with those found in HEK-293 T cells (Figure 4F and 4G). Additional file 2: Figure S3 showed that K562 cells were in good condition and displayed high transfection rate.

**Figure 5:**
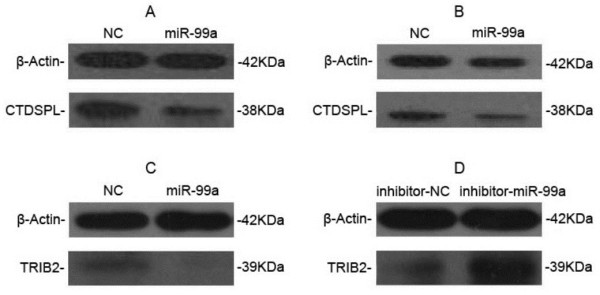
**Role of miR-99a in expressions of CTDSPL and TRIB2 proteins in HL60 or K562 cells.** The expression of CTDSPL in HL60 **(A)** or K562 **(B)** cells transfected with 100 nM miR-99a mimics or NC duplex, respectively. The expression of TRIB2 in K562 cells transfected with 100 nM miR-99a mimics or NC duplex **(C)**, or 100 nM inhibitor-miR-99a or inhibitor-NC **(D)**, respectively.

In addition, the ability of miR-99a to regulate the endogenous CTDSPL and TRIB2 proteins was tested. Results showed that the expression of CTDSPL protein decreased in HL60 or K562 cells transfected with miR-99a and cultured for 4 days, compared with the cells tranfected with NC duplex (Figure 5A and 5B). Similarly, the expression of TRIB2 protein also decreased in K562 cells transfected with miR-99a (Figure 5C), however, increased significantly in the cells transfected with inhibitor-miR-99a compared with those transfected with inhibitor-NC (Figure 5D). Collectively, it is strongly suggested that miR-99a targets and suppresses CTDSPL and TRIB2 [17-19].

### MiR-99a represses expression of CTDSPL and TRIB2 proteins in most clinical samples from AML patients

**Figure 6:**
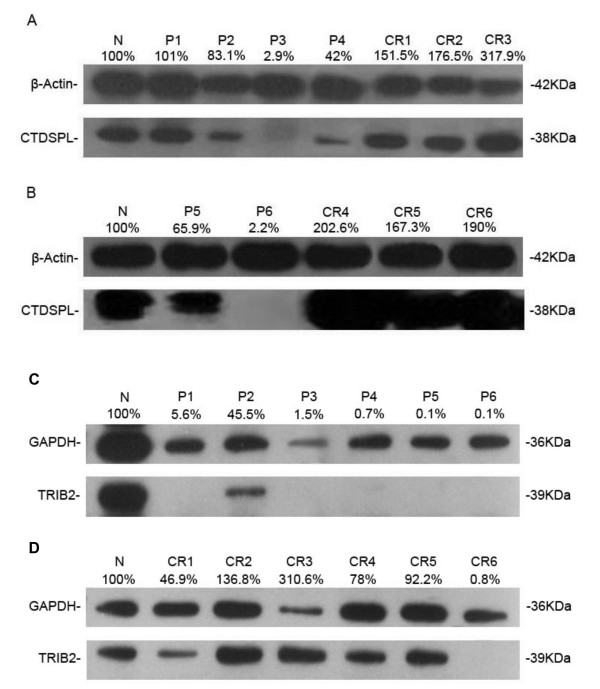
**MiR-99a represses expression of CTDSPL and TRIB2 proteins in most clinical samples from AML patients.** Western blot showed that the expression level of CTDSPL protein in patients with AML-M3 **(A)** and AML-M2 **(B)** and the expression level of TRIB2 protein in patients with AML-M3 **(C, D)** were relatively high in children in control group (N) and most of the patients in hematological CR; however, the level decreased in most of the patients before treatment (P).

Based on the experimental results mentioned above, we assumed that the expressions of CTDSPL and TRIB2 decrease in AML patients at diagnosis and increase in those in CR, which is contrary to the expression of miR-99a. A total of 38 bone marrow samples that had sufficient cell material available were used to determine the expression of CTDSPL by western blot, which were from 4 controls and 34 AML patients with M2 and M3 (15 at diagnosis, 19 in hematological CR, all of which were not in pairs). As expected, the expression level of CTDSPL was relatively high in control group and in 58% of the patients with CR; however, it was down-regulated in 86.6% of the patients at diagnosis (Figure 6A, 6B and Additional file 2: Figure S4). In addition, a total of 30 samples that had sufficient cell material available were used to determine the expression of TRIB2 protein, including 2 controls and 28 AML patients with M2 and M3 (15 at diagnosis and 13 with hematological CR, not in pairs). Results showed that the expression level of TRIB2 protein was relatively high in control group and in 69.2% of the patients with CR, while the expression level decreased in 73.7% of the patients at diagnosis (Figure 6C, 6D and Additional file 2: Figure S5). All these further support that miR-99a targets CTDSPL and TRIB2 genes.

In conclusion, our study indicates that miR-99a may play an oncogenic role by targeting the tumor suppressors CTDSPL and TRIB2 in most pediatric myeloid leukemia.

## Discussion

Leukemia is the commonest childhood malignant disease. With the rapid development of modern combination chemotherapy and hematopoietic stem cell transplantation, 5-year event-free survival for pediatric acute lymphoblastic leukemia (ALL) has been improved to rates as high as 80% [20,21]. However, the prognosis of pediatric AML is still poor, with long-term survival rates of about 50% to 65% [22]. The overall survival of CML was recently reported to be up to 80% at 8 years of follow-up in respondent patients due to the introduction of imatinib (a tyrosine kinase inhibitor) [23], there still remains a subset of patients who fail the treatment. It is therefore of significance to clarify the molecular mechanisms of these two diseases for further improving survival rate. For a long time, the pathogenesis researches of AML and CML mainly focus on chromosome abnormalities and protein coding genes. Recently, more and more studies indicated that abnormal expressions of relevant miRNAs may promote tumors. Their abnormal expressions are closely related to the incidence, development, treatment response and prognosis of leukemia [11,24-28]. Although some miRNA expression signatures associated with types and cytogenetics of leukemia have been addressed, there has been no report on miR-99a expressional and functional study in pediatric AML and CML so far.

In this study, we found that the expression of miR-99a increased significantly not only in childhood patients with AML (M1-M5) but also in those with CML, decreased obviously in CR patients with these two myeloid leukemias, and increased again in relapsed patients with AML-M2 analyzed. Furthermore, MTT assay showed that the proliferation of K562 and HL60 cells was effectively promoted by miR-99a, and apoptosis experiment demonstrated that the apoptosis of K562 and HL60 cells was suppressed by miR-99a. These results illustrate that miR-99a may function as an oncogene, which contributes to the generation and development of both AML and CML in children. Finally, dual-luciferase reporter transfection assay and western blot analysis on clinical samples and leukemia cell lines further supported that miR-99a played a potential oncogene role by targeting CTDSPL and TRIB2 in most pediatric myeloid leukemia patients.

CTDSPL gene exhibits tumor suppressor gene activity. It has been reported that CTDSPL protein plays the role of phosphatase, regulating cells growth and differentiation, and expresses significantly low in major epithelial malignancies [15]. In leukemia cell lines and 24% of patients with acute lymphoblastic leukemia, CTDSPL promoter is highly methylated, which promotes the occurrence of leukemia [29]. A study further revealed that RBSP3, also denoted as HYA22 and CTDSPL, is involved in the regulation of cell growth and differentiation, and frequent mutations in this gene are detected in human hematopoietic cell lines [16]. The tumor suppressor property of CTDSPL is related to its ability to remove the phosphate group from serine 807 and 811, and induce the formation of the RB-E2F1 complex [30]. The 'RB’ pathway has a critical role in both cell physiology and tumorigenic transformation via distinct molecular mechanisms.

TRIB2, like some other genes, has distinctive roles in different biological conditions. On the one hand, TRIB2, a pseudokinase, may function as an oncogene and cooperates with HoxA9 to accelerate the onset of AML in mice by binding COP1 and C/EBP-α and leading to degradation of C/EBP-α [31-33]. On the other hand, TRIB2 may serve as a tumor suppressor gene. A resent report showed that forced expression of PITX1 in JURKAT cells prompted deregulation of genes involved in T-cell development including TRIB2. Leukemic activation of PITX1 was observed in a subset of early-staged T-ALL by inhibiting T-cell development [34]. TRIB2 is also a pro-apoptotic molecule and activates Bax gene to induce apoptosis in hematopoietic cells through degradation of MCL-1 [35]. Its tumour suppressor activity may be abrogated in a proportion of AML patients, which may lead to enhanced cell survival and therefore contribute to pathogenesis of the disease [36].

MiR-99a has been addressed to be involved in the tumorigenesis of several cancers. MiR-99a may play different roles in different tumors. Wong and Wszolek reported that miR-99a expresses notably high in untreated patients with tongue squamous cell carcinoma and non-invasive urothelial cancer, suggesting that miR-99a exhibits oncogenic activity [37,38]. However, Yamada et al. addressed that the expression of miR-99a deceased in onset patients with lung cancer, implying that miR-99a may behave as a tumor suppressor gene [39]. In this study, we find that miR-99a plays a potential oncogenic role in pediatric AML and CML through targeting CTDSPL and TRIB2. All the above studies elucidate that the roles of certain miRNAs are completely distinctive in different biological conditions, which is also supported by some other studies [18,19,40-43]. All these highlight the importance of thoroughly studying and comprehensively expounding the expression and function of miRNAs in different tumors as well as the same tumor in adults and children.

It is noted that in this study there was a relatively larger number of clinical samples which were tested for the expressions of miR-99a, CTDSPL and TRIB2 compared with previous studies. The results revealed that miR-99a may target CTDSPL and TRIB2 more convincingly from a statistical point of view. However, the results also showed that CTDSPL or TRIB2 protein and miR-99a exhibited opposite expression trends in most the patients, but not in all the patients, indicating that CTDSPL and TRIB2 genes are not the only target genes of miR-99a. Therefore, there may be other potential undiscovered target genes, which is in line with our present knowledge. As a single miRNA could regulate several different genes and the same gene could also be regulated by several miRNAs [44,45], the regulation of miRNA forms a complex network. The complicated interactions of relevant miRNAs contribute to the occurrence and development of leukemia.

## Conclusions

This is the first report to demonstrate the expression and function of miR-99a in childhood AML and CML. MiR-99a targets CTDSPL and TRIB2, and regulates their expressions in most childhood AML and CML, which may reveal a new post-transcriptional mechanism of regulation in the myeloid lineage. Additionally, this study suggests that there might be some common biological pathways involved in disease development of AML and CML although they are two clinically different myeloid leukemias and miR-99a could be a common therapeutic target for the treatment of these two myeloid leukemias.

## Abbreviations

AML: Acute myeloid leukemia; CML: Chronic myeloid leukemia; qRT-PCR: Quantitative real-time reverse transcriptase PCR; miRNA: MicroRNA; CTDSP: CTD (carboxy-terminal domain, RNA polymerase II, polypeptide A) small phosphatase-like; TRIB2: Tribbles homolog 2 (Drosophila); MCL-1: Myeloid cell leukemia sequence 1 (BCL2-related).

## Competing interests

The authors declare that they have no competing interests.

## Authors’ contributions

LDZ performed experiments, analyzed the data and wrote the manuscript; XJL, YNL JW and XLZ performed a part of western blot; ZYK and LBH provided samples for the analysis; YQC, HZ and XQL designed experiments and edited the manuscript. All authors critically reviewed the manuscript. All authors read and approved the final manuscript.

## Supplementary Material

Additional file 1: Table S1 Characteristics of patients with AML. **Table S2.** Characteristics of patients with CML. **Table S3.** Primer sequence of miR-99a. **Table S4.** Primer sequences for vector construction. Click here for file

Additional file 2: Figure S1 HL60 cells transfected with negative control labeled with 100 nM FAM fluorescent by Liposome (A) and fluorescence in the HL60 cells (B) at 36 h after transfection; K562 cells transfected with the negative control (C) and fluorescence in the K562 cells (D) at 36 h after transfection. **Figure S2.** HEK-293 T cells transfected with PCD6.2 vector (A) and fluorescence in the HEK-293 T cells (B) at 28 h after transfection. **Figure S3.** K562 cells transfected with PCD6.2 vector (A) and fluorescence in the K562 cells (B) at 28 h after transfection. **Figure S4.** MiR-99a represses expression of CTDSPL proteins in most clinical samples from AML patients. In patients with AML-M3 (A, B) and AML-M2 (C, D), western blot showed that the expression level of CTDSPL protein was relatively high in children in control group (N) and most of the patients in hematological CR; however, the level decreased in most of the patients before treatment (P). **Figure S5.** MiR-99a represses expression of TRIB2 proteins in most clinical samples from AML patients. In patients with AML-M2 (A-C), western blot showed that the expression level of TRIB2 protein was relatively high in children in control group (N) and most of the patients in hematological CR; however, the level decreased in most of the patients before treatment (P). Click here for file
