# Supplementary material for: MiR-99a may serve as a potential oncogene in pediatric myeloid leukemia
Source: Cancer Cell Int. 2013 Nov 5;13:110. doi: 10.1186/1475-2867-13-110 (PMC4176743; doi:10.1186/1475-2867-13-110)
Supplement: Additional file 1: Table S1 — Characteristics of patients with AML. Table S2. Characteristics of patients with CML. Table S3. Primer sequence of miR-99a. Table S4. Primer sequences for vector construction. [file 1475-2867-13-110-S1.doc]

| **Table 1. Characteristics of patients with AML.** | | | |
| --- | --- | --- | --- |
| **Stage of the disease** | **Characteristics** | **Median (range)** | **No. (%)** |
| **Before therapy** | **Age at diagnosis, yr** | 8 (3-12) | 41 |
|  | **Sex** |  |  |
|  | Male |  | 25 (61.0) |
|  | Female |  | 16 (39.0) |
|  | **WBC count, ×10^9^/L** | 19.8 (2.8-62.3) |  |
|  | Less than 10 |  | 17 (41.5) |
|  | 10-50 |  | 15 (36.5) |
|  | 50 or higher |  | 9 (22.0) |
|  | **FAB** |  |  |
|  | M1 |  | 6 (14.6) |
|  | M2 |  | 17 (41.4) |
|  | M3 |  | 10 (24.4) |
|  | M4 |  | 4 (9.8) |
|  | M5 |  | 4 (9.8) |
|  | **AML1/ETO (M2)** |  |  |
|  | Positive |  | 10 (58.8) |
|  | Negative |  | 3 (17.7) |
|  | Not examined |  | 4 (23.5) |
|  | **PML/RARA (M3)** |  |  |
|  | Positive |  | 9 (90.0) |
|  | Negative |  | 1 (10.0) |
| **Complete remission**^&^  **Relapse** |  |  | 23 (85.2)  4 (14.8) |
| & With at least three years of clinical follow-up. | | | |

**.**

| **Table 2. Characteristics of patients with CML.** | | |
| --- | --- | --- |
| **Characteristics** | **Median (range)** | **No. (%)** |
| **Age at diagnosis, yr** | 8.9(5-12) | 8 |
| **Sex** |  |  |
| Male |  | 5 (62.5) |
| Female |  | 3 (37.5) |
| **WBC at diagnosis, ×10^9^/L** | 78.45 (29.3-112.8) |  |
| 100 or higher |  | 2 (25.0) |
| 50-100 |  | 5 (62.5) |
| Less than 50 |  | 1(12.5) |
| **BCR-ABL** (Positive) |  | 8(100%) |
| **WBC in CR, ×10^9^/L** |  |  |
| Higher than 10 |  | 2(50.0) |
| Less than 10 |  | 2(50.0) |
| **Stage of the disease** |  |  |
| Before therapy |  | 4(50.0) |
| Complete remission ^&^ |  | 4(50.0) |
| & With at least three years of clinical follow-up. | | |

| **Table 3. Primer sequence of miR-99a.** | |
| --- | --- |
| **Primer Name** | **Sequence 5'to3'** |
| miR-99a-F | CGTAGCATCCTTAGAACTCAGC |
| miR-99a-R | CCGCTCGAGGAACTATTGTTGAACGGCACT |
| PCD6.2-S | GCTAAGCACTTCGTGGCCGTC |
| PCD6.2-R | TTGTACAAGAAAGCTGGGTC |
| F: forward primer; R: reverse primer; | |

| **Table 4.** Primer sequences for vector construction. | |
| --- | --- |
| **Primer Name** | **Sequence 5'to3'** |
| CTDSPL-UTR-F | GATCCGACGAATGTTTGGGGTTTATGTTTTTTAAGGTAAATACGGGTATTGTTTTTAAC |
| CTDSPL-UTR-R | TCGAGTTAAAAACAATACCCGTATTTACCTTAAAAAACATAAACCCCAAACATTCGTCG |
| CTDSPL-M-F | GATCCGACGAATGTTTGGGGTTTATGTTTTTTAAGGTAAATTGTTTTTAAC |
| CTDSPL-M-R | TCGAGTTAAAAACGTATTTACCTTAAAAAACATAAACCCCAAACATTCGTCG |
| TRIB2-UTR-F | TCGAG GTCACATTGTGATGTATTAAGCCAGTACTTCAATTACGGGTTGACTTGGGATGC |
| TRIB2-UTR-R | GGCCGCATCCCAAGTCAACCCGTAATTGAAGTACTGGCTTAATACATCACAATGTGACC |
| TRIB2-M-F | TCGAG GTCACATTGTGATGTATTAAGCCAGTACTTCAATTGACTTGGGAT GC |
| TRIB2-M-R | GGCCGC ATCCCAAGTCAATTGAAGTACTGGCTTAATACATCACAATGTGACC |
| PGL3-S | GGAAAACTCGACGCAAGAAA |
| PGL3-R | ACCTCCCCCTGAACCTGAAACA |
| Psi-check2-S | GAGGACGCTCCAGATGAAATGGG |
| Psi-check2-R | CGCCTCCGAATGAGAGTGTTTCG |
| F: forward primer; R: reverse primer. | |

朗读

显示对应的拉丁字符的拼音
