# Supplementary material for: MiR-99a may serve as a potential oncogene in pediatric myeloid leukemia
Source: Cancer Cell Int. 2013 Nov 5;13:110. doi: 10.1186/1475-2867-13-110 (PMC4176743; doi:10.1186/1475-2867-13-110)

**Additional file 1 figure legends**

Fig. 1. HL60 cells transfected with negative control labeled with 100nM FAM fluorescent by Liposome (A) and fluorescence in the HL60 cells (B) at 36h after transfection; K562 cells transfected with the negative control (C) and fluorescence in the K562 cells (D) at 36h after transfection.

Fig. 2. HEK-293T cells transfected with PCD6.2 vector (A) and fluorescence in the HEK-293T cells (B) at 28h after transfection.

Fig. 3. K562 cells transfected with PCD6.2 vector (A) and fluorescence in the K562 cells (B) at 28h after transfection.

Fig. 4. MiR-99a represses expression of CTDSPL proteins in most clinical samples from AML patients. In patients with AML-M3 (A, B) and AML-M2 (C, D), western blot showed that the expression level of CTDSPL protein was relatively high in children in control group (N) and most of the patients in hematological CR; however, the level decreased in most of the patients before treatment (P).

Fig. 5. MiR-99a represses expression of TRIB2 proteins in most clinical samples from AML patients. In patients with AML-M2 (A-C), western blot showed that the expression level of TRIB2 protein was relatively high in children in control group (N) and most of the patients in hematological CR; however, the level decreased in most of the patients before treatment (P).

Fig. 1.


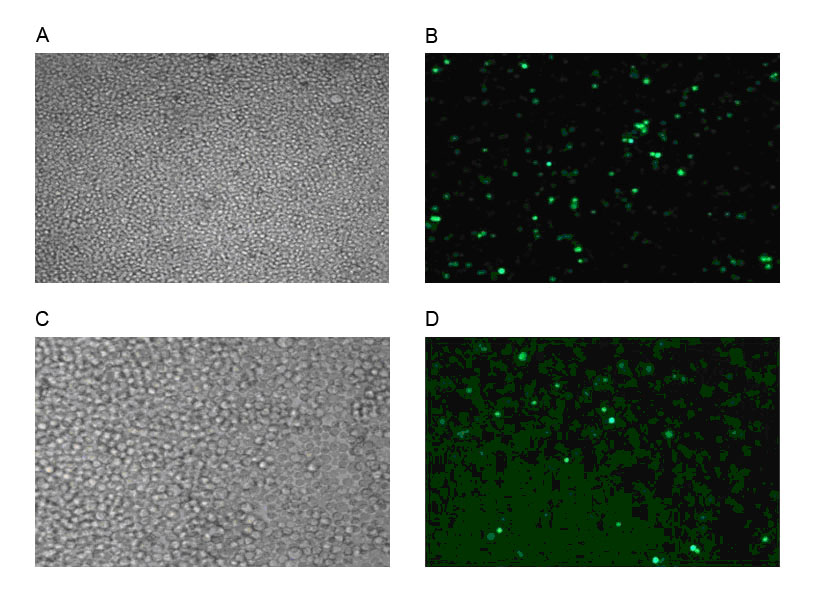


Fig. 2.


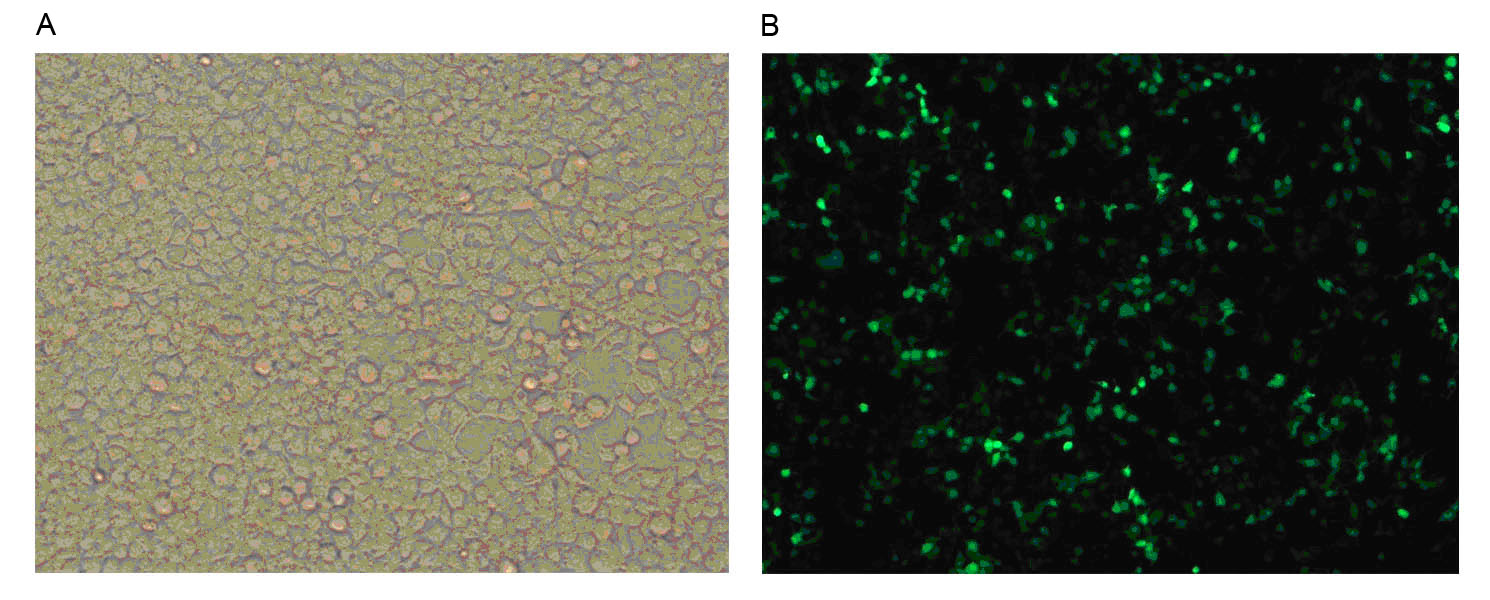


Fig. 3.


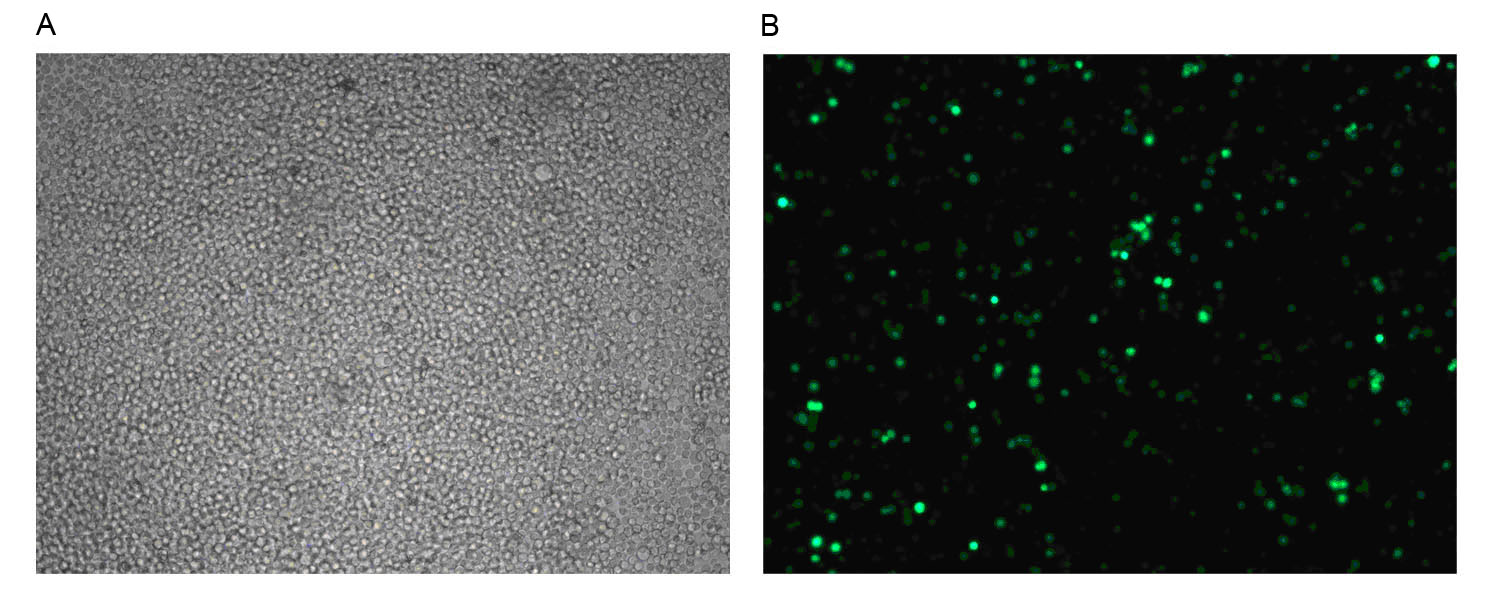


Fig. 4.


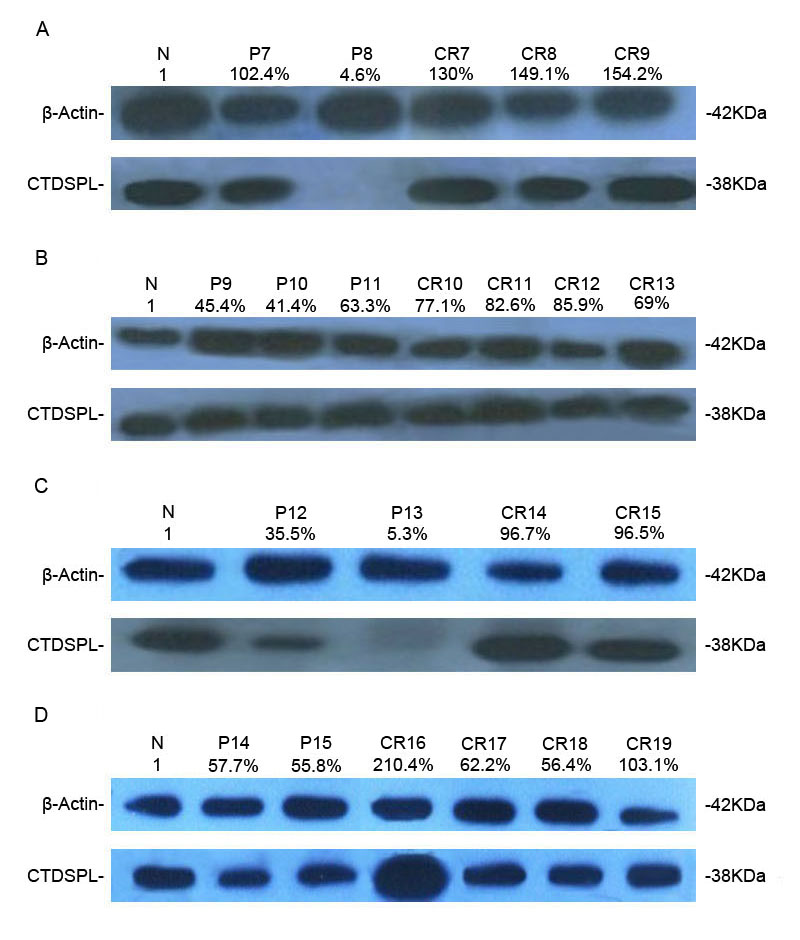


Fig. 5.


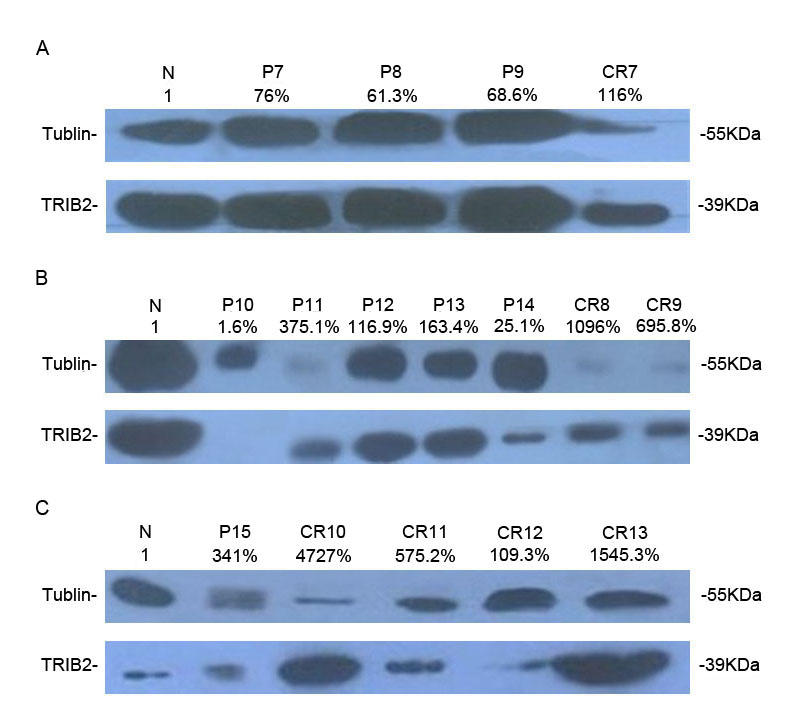

Supplement: Additional file 2: Figure S1 — HL60 cells transfected with negative control labeled with 100 nM FAM fluorescent by Liposome (A) and fluorescence in the HL60 cells (B) at 36 h after transfection; K562 cells transfected with the negative control (C) and fluorescence in the K562 cells (D) at 36 h after transfection. Figure S2. HEK-293 T cells transfected with PCD6.2 vector (A) and fluorescence in the HEK-293 T cells (B) at 28 h after transfection. Figure S3. K562 cells transfected with PCD6.2 vector (A) and fluorescence in the K562 cells (B) at 28 h after transfection. Figure S4. MiR-99a represses expression of CTDSPL proteins in most clinical samples from AML patients. In patients with AML-M3 (A, B) and AML-M2 (C, D), western blot showed that the expression level of CTDSPL protein was relatively high in children in control group (N) and most of the patients in hematological CR; however, the level decreased in most of the patients before treatment (P). Figure S5. MiR-99a represses expression of TRIB2 proteins in most clinical samples from AML patients. In patients with AML-M2 (A-C), western blot showed that the expression level of TRIB2 protein was relatively high in children in control group (N) and most of the patients in hematological CR; however, the level decreased in most of the patients before treatment (P). [file 1475-2867-13-110-S2.doc]
